# The Power of Suggestion: Subjective Satiety Is Affected by Nutrient and Health-Focused Food Labelling with No Effect on Physiological Gut Hormone Release

**DOI:** 10.3390/nu14235100

**Published:** 2022-12-01

**Authors:** Sinead Watson, Hannah O’Hara, Dharsshini Reveendran, Christopher Cardwell, Kevin G. Murphy, Tony Benson, Moira Dean, Jayne V. Woodside

**Affiliations:** 1 Centre for Public Health, School of Medicine, Dentistry and Biomedical Sciences, Queen’s University Belfast, Belfast BT12 6BA, UK; 2 Department of Metabolism, Digestion and Reproduction, Faculty of Medicine, Imperial College London, London W12 0NN, UK; 3 Institute for Global Food Security, School of Biological Sciences, Queen’s University Belfast, Belfast BT9 5AG, UK

**Keywords:** food labelling, health claims, nutrition, gut hormones, appetite, hunger

## Abstract

Health claims on food labelling can influence peoples’ perception of food without them actually eating it, for example driving a belief that a particular food will make them feel fuller. The aim of this study was to investigate whether nutrient and health claims on food labelling can influence self-reported, and physiological indicators of, satiation. A total of 50 participants attended two visits where they were asked to consume a 380 kcal breakfast (granola and yogurt) labelled as a 500 kcal ‘indulgent’ breakfast at one visit and as a 250 kcal ‘sensible’ breakfast at the other. The order of the breakfast descriptions was randomly allocated. Participants were unaware that the two breakfasts were the same product and that only the food labels differed. At each visit blood samples were collected to measure gut hormone levels (acylated ghrelin, peptide tyrosine-tyrosine and glucagon-like peptide-1) at three time points: 20 min after arrival (baseline), after 60 min (anticipatory, immediately prior to consumption) and after 90 min (post-consumption). Visual analogue scales measuring appetite (hunger, satiety, fullness, quantity and desire to eat) were completed prior to each sample. Between 60 and 90 min, participants consumed the breakfast and rated its sensory appeal. Participants reported a higher mean change in self-reported fullness for the ‘indulgent’ than the ‘sensible’ breakfast from anticipatory to post-consumption (mean difference: 7.19 [95% CI: 0.73, 13.6]; *p* = 0.030). This change was not observed for the other appetite measures at the other time points or gut hormone levels. This study suggests that nutrient and health claims on food labels may influence satiation as measured by self-reported fullness. It also suggests that the observed differences in satiety scores are not due to changes in the main appetite regulating gut hormones, but are more likely centrally mediated. More high-quality trials are required to confirm these findings.

## 1. Introduction

The control of energy intake from food is complex and is governed by the interaction of both metabolic and emotional/cognitive regulatory systems. Peoples’ food preferences and enjoyment of food appear to be subject to suggestibility from visual and cognitive cues. For example, people prefer the taste of a branded beverage when consumed from a brand-named cup [1]. In addition, functional magnetic resonance imaging (fMRI) shows that there is increased activity in areas of the brain involved in emotional processing when the beverage is consumed after being shown a brand image compared to when consumed with no image being shown [1]. Furthermore, when a low calorie drink is ingested following the spoken verbal descriptor ‘treat’, there is increased activity in the hypothalamus and midbrain, as measured by fMRI, compared to when it is described as ‘healthy’ [2]. In addition, labelling and describing food items with taste-promoting descriptors increases selection of the food item and increases taste scores following consumption compared to when the same food is promoted as healthy [3]. Indeed foods felt to be unhealthy are perceived to be tastier [4].

Endeavouring to reduce rates of overweight and obesity, public health policies commonly aim to educate the public regarding making healthy food choices [5,6]. Food marketers have capitalised on the public’s desire to eat ‘healthily’, health and nutrient claims which imply that food have particular beneficial health or nutritional properties are often used to boost sales. Indeed, surveys have shown that people are willing to pay more for healthy food [7]. Using health claims on food labelling has been shown to influence peoples’ perception of food without them actually eating it, for example, driving the belief that a particular food will make them feel more full [8,9]. Placing nutritional content on food labelling of unhealthy food using health star ratings or multiple traffic lights, for example, can restrain a person’s selection of portion size [10]. Perceived macronutrient content has also been demonstrated to influence subsequent energy intake, with participants consuming more at a test meal after eating a preload labelled as ‘low fat’ comparted to participants who consumed the same preload labelled as ‘high fat’ [11]. However, studies evaluating the effects of food labelling on appetite have produced variable results. A study by Chambers et al. (2013) found that the sensory qualities of a smoothie preload influenced appetite ratings and subsequent food intake, but labelled messages regarding the satiating properties of the product did not [12]. A study by Yeomans et al. (2001) demonstrated that soup preloads labelled as ‘high fat’ were rated as being more pleasant and creamy than those labelled as ‘low fat’, regardless of actual fat content. However, energy intake at a subsequent test meal was unaffected by the preload label. Instead, energy intake was influenced by the actual fat content, with a higher fat preload associated with lower subsequent energy intake [13]. Studies have also demonstrated that claims such as ‘low-fat’ and ‘low sugar’ can actually encourage people to eat more, perhaps by a reduction in the anticipated guilt associated with eating the ‘healthy’ option, or by increasing the amount of food felt necessary to satiate due to calorie underestimation [14,15,16].

There is emerging evidence that the influence of product claims can extend beyond perception to actually influence the physiological indicators of satiety. Cassidy et al. (2012) conducted a study where participants consumed oral liquid and solid preloads which, following observation of a falsified demonstration, they were led to believe either formed liquids or solids in the stomach when in fact all preloads either remained liquid or quickly turned to liquid on consumption. They found that preloads which were either liquid, or perceived to be liquid once in the stomach, elicited greater postprandial hunger and lower fullness sensations, and attenuated insulin and glucacon-like peptide 1 release compared to those consumed as a solid, or perceived to turn to solid in the stomach [17]. Ghrelin is an orexigenic peptide synthesised in the stomach and is the only gastrointestinal hormone known to stimulate appetite. A study by Crum et al. (2011) demonstrated that when participants believed they were consuming an ‘indulgent’ product versus one labelled as ‘sensible’, there was a significantly deeper decline in the levels of ghrelin, despite the products being nutritionally identical [18]. However, in this study there were no reported differences in subjective hunger scores after consuming the differently labelled products, and the study did not assess any subjective measures of satiety. The role of food descriptions in promoting satiety and the mechanisms underlying this are unclear, but if these mechanisms could be further explored and the effect on satiety could be replicated, it could have implications for food labelling and marketing, as well as public health strategy. The aim of the current study was therefore to investigate whether the consumption of a 380 kcal yoghurt and granola breakfast item resulted in different subjective satiety scores or gastrointestinal hormone levels that mediate appetite, depending on whether the product was labelled as an ‘indulgent’ 500 kcal product (high in fat and sugar) or a ‘sensible’ 250 kcal one (low in fat and sugar). To achieve this, self-reported appetite and the circulating concentrations of the gastrointestinal hormone acylated ghrelin were measured. Peptide tyrosine-tyrosine (PYY) and glucagon-like peptide-1 (GLP-1) are anorectic gut hormones released into the circulation post-prandially in response to luminal nutrients, signalling satiety and promoting meal cessation. Circulating concentrations of these hormones were also measured.

## 2. Materials and Methods

### 2.1. Study Design

The study design was an adaptation of Crum et al. [18] and a similar sample size was used. As the study objectives were exploratory, and no variability data was available from Crum et al. a formal power calculation was not conducted. Eligible participants were invited to attend the Northern Ireland Clinical Research Facility (NICRF), on two separate occasions, with an interval of one week between visits. All visits occurred in the morning following an overnight fast and lasted approximately two hours. Participants were asked to attend their second visit at the same time as their first visit the week before, and were assessed individually. At the first visit participants were told that two granola, yoghurt and blackcurrant compote breakfast products with different energy and nutrient contents have been developed by researchers at Queen’s University Belfast (QUB), and that they would be asked to consume one breakfast product at the first visit and the other breakfast product at the second visit. 

**Figure 1:**
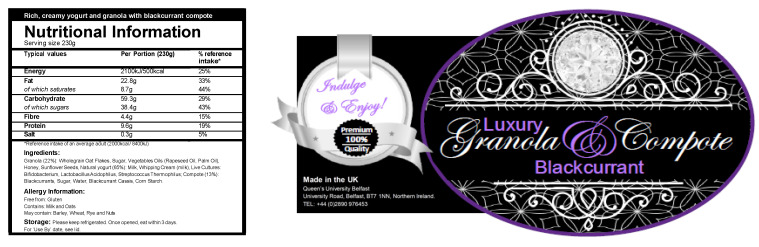
‘Indulgent’ granola and yoghurt label.

**Figure 2:**
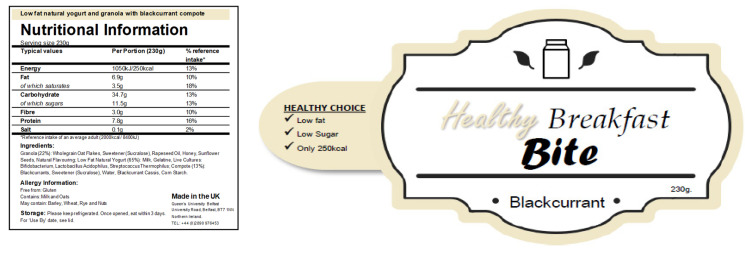
‘Sensible’ granola andyoghurt label.

One of the breakfast products was presented as the “indulgent” option and was described as having 500 kcal per serving (230 g) and being high in fat and sugar (Figure 1), while the other breakfast product was presented as the “sensible” option, and was described as having 250 kcal per serving (230 g) and being low in fat and sugar (Figure 2) The actual content of the breakfast products was identical (nutritional information shown in Table 1). The participants were told that the aim of the study was to evaluate whether the two different granola, yoghurt and compote breakfast products tasted similar, and to examine the body’s response to the different options. The participants were unaware that the two breakfast products were the same 380 kcal product, and that only the food labels differed. The order of the breakfast products, ‘indulgent’ or ‘sensible’ was randomly allocated, using block randomisation (block size = 4), to ensure the presentation of test conditions was counterbalanced to neutralise possible learning effects. 

**Figure 3:**
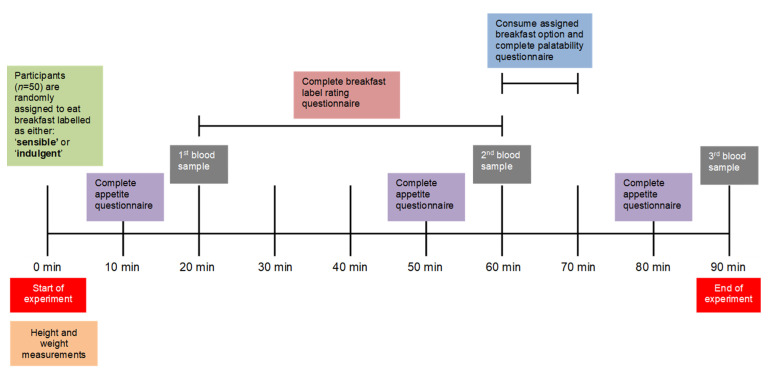
Experimental timeline.

Figure 3 summarises the experimental timeline for each visit. At each visit, 6 mL blood samples were collected at three time-points: after a 20 min rest period (baseline), after 60 min (anticipatory) and after 90 min (post-consumption). Participants were asked to complete self-reported appetite measures 10 min prior to each blood sample. During the first interval, between blood sampling (between 20 and 60 min), participants were asked to rate the breakfast label based on its appearance and perceived healthiness, and during the second interval (between 60 and 90 min) participants were asked to consume the entire breakfast product within 10 min while rating the breakfast’s sensory appeal.

### 2.2. Participants

Participants were recruited from May to September 2017 using convenience and snowball sampling. The study was promoted by placing posters in public places, by advertising on the NICRF website, and by an intranet advertisement via QUB staff updates. Other recruitment methods included word-of-mouth personal referrals from existing participants and QUB researchers. Participants were eligible if they were aged between 18 and 64 years, and were excluded if they were pregnant; had diabetes (type 1 or 2); reported taking medication that could affect their appetite, taste or smell; and if they reported having dietary restrictions to wheat, nuts, eggs or dairy products. The Queen’s University Belfast School of Medicine, Dentistry and Biomedical Sciences Research Ethics Committee granted ethical approval. All participants provided written informed consent. All participants were informed about the study’s element of deception once all the study visits were completed. Participants were paid an honorarium of £30 to cover time and travels costs if they attended both study visits.

### 2.3. Data Collection and Analysis

#### 2.3.1. Socio-Demographic

At the first visit participants were asked to complete a questionnaire that collected socio-demographic information such as age, gender, education level, occupation, marital status and whether they were an urban or rural dweller. The questionnaire also collected medical and lifestyle information such as smoking status, health history, dietary restrictions, medication and nutritional supplement use. Socio-economic status (SES) was based on current occupation and was determined by the National Statistics Socio-Economic Classification System (NSSEC) [19].

#### 2.3.2. Anthropometry

Height (m) and weight (kg) were measured using a stadiometer and calibrated scales, respectively, at the first visit. Body mass index (BMI) was calculated as weight divided by height squared.

#### 2.3.3. Restrained Eating

Restrained eating patterns have been associated with higher ghrelin levels [20] as well as a tendency to eat more when offered supposedly ‘healthy’ food [21]. A sub-scale of the Dutch Eating Behaviour Questionnaire (DEBQ) to assess dietary restraint [22] was also completed at the first visit. Scoring of the DEBQ involved adding the scores of the 10 items (5 point Likert Scale) to produce a raw score, then dividing it by 10 to compute a scale score for restrained eating behaviour. 

#### 2.3.4. Self-Reported Appetite

Visual Analogue Scales (VAS), 100 mm in length with words anchored at each end, expressing the most negative rating (0) and the most positive (100), were used to assess hunger, satiety, fullness, prospective food consumption and desire to eat [23,24] at each visit. Participants were required to complete these scales 10 min prior to each blood sample collection. 

#### 2.3.5. Breakfast Label Rating

During the first interval, between the collection of blood samples (between 20 and 60 min), participants were asked to rate the food product, based on the (misleading) label, on its appearance, perceived healthiness and its overall appeal at each visit. Responses were assessed using 100 mm VAS as described above. Participants were also asked how often (always, often, occasionally, rarely and never) they read the nutritional and health claim information on food labels.

#### 2.3.6. Palatability Rating

While consuming the breakfast, participants were asked to rate the breakfast based on its visual appeal, smell, taste, overall palatability, enjoyment and if they felt healthy when consuming the breakfast. Responses were assessed using 100 mm VAS as described above.

#### 2.3.7. Gastrointestinal Hormones

At each visit a blood sample was collected at three time-points with three separate blood draws (Figure 3): after a 20 min rest period (baseline), after 60 min (anticipatory) and after 90 min (post-consumption). A protease inhibitor (AEBSF) was added to the blood samples immediately after collection. Samples were centrifuged within 15 min of collection, and plasma stored at −80 °C until batch analysed. 

Three types of gut hormones were measured in plasma samples: acylated ghrelin, peptide tyrosine-tyrosine (PYY) and glucagon-like peptide-1 (GLP-1). Acylated ghrelin is secreted from endocrine cells of the stomach when there is a low energy intake or an empty stomach, while PYY and GLP-1 are secreted postprandially by L-cells of the ileum and colon and are reduced during periods of fasting.

Samples were analysed for acylated ghrelin by an ELISA method (BioVendor, Czech Republic. Cat No: RA194062500R) using an automated Triturus analyser at QUB. The limit of detection (LOD) for the assay was 1.2 pmol/L with an intra-assay coefficient of variation (CV) of 5.9%. Total GLP-1 and total PYY were measured using established in-house radioimmunoassay [25,26] at Imperial College London. The LODs for the GLP-1 and PYY assays were 7.5 pmol/L and 2.5 pmol/L, with intra-assay CVs of 7.1% and 9.6%, respectively. 

### 2.4. Statistical Analysis

All analyses were performed using SPSS for Windows version 25.0 (SPSS Inc., Chicago, IL, USA). The analytical plan was pre-specified in the study protocol. Descriptive statistics (means [SD] and frequencies [%]) were used to summarise the characteristics of the sample. Paired sample *t*-tests were performed on ratings of appeal and perceived healthiness, as well as on palatability (appearance, taste, smell, overall palatability and enjoyment), to investigate the within-group effect of the breakfast description (indulgent vs. sensible) on these measures. Hills and Armitage analysis was then used for a two-period a crossover design using SPSS v17.0 for Windows (SPSS Inc., Chicago, IL, USA) and Excel (Windows 2013) [27]. The change in self-reported appetite scores (hunger, satiety, fullness, prospective food consumption and desire to eat) and gut hormones (acylated ghrelin, PYY and GLP-1) from the time points (T): (1) baseline (BL) to anticipatory (ANT), (2) anticipatory (ANT) to post-consumption (POST), and (3) baseline (BL) to post-consumption (POST), were computed for both breakfast conditions and for both visits. To investigate the effect of the breakfast condition (indulgent vs. sensible) on change in self-reported appetite and the gut hormones at the aforementioned time points, paired sample *t*-tests were conducted. Differences in mean were adjusted for the period, and 95% confidence intervals were calculated.

Participants were categorised into two groups according to their reported level of restrained eating behaviour (high vs. low) using median-split (median DEBQ score: 2.7 [IQ: 2.2–3.4]). The above analyses were repeated separately for each of these groups for the self-reported appetite scores and gut hormone levels. 

## 3. Results

### 3.1. Sample Characteristics

A total of 50 participants aged between 19 and 60 years (mean ± SD: 30.1 ± 10.4 years) were recruited and attended the first visit. Twenty-three participants were recruited by the various advertising methods described above, and the rest of the sample (n = 27) were recruited by word-of-mouth personal referrals. Two participants did not attend the second visit owing to issues with blood sampling at the first visit; three participants had one blood sample missing from at least one of the visits; seven participants did not have results for acylated ghrelin due to failure of the assay; four participants’ PYY levels were below the LOD; the PYY assay failed for one participant; and one participant’s GLP-1 level was below the LOD. Therefore, in total 48 participants had completed all the self-reported appetite questionnaires, 38 participants had complete acylated ghrelin results, 40 participants had complete PYY results and 44 participants had complete GLP-1 results.

Table 2 shows the characteristics of the sample. The sample consisted mostly of QUB undergraduate/postgraduate students (62%) and staff members (32%). All participants were non-smokers and 38% of the participants were classified as overweight/obese. Sixty percent of the participants reported reading nutritional information on food labels often/always, while 44% reported reading health claim information often/always. 

### 3.2. Rating of Breakfast Labels and Palatability

Table 3 shows the mean differences in rating scores for the breakfasts’ packaging and palatability according to breakfast condition (indulgent vs. sensible). Prior to consumption participants rated the “indulgent” breakfast packaging as more appealing (mean difference: 17.29 [95% CI: 11.6, 23.0]; *p* < 0.001) but believed it was less healthy (mean difference: −24.78 [95% CI: −31.62, −17.94]; *p* < 0.001) than the “sensible” breakfast. Similar results were found during consumption when participants completed the palatability questionnaire. Participants rated the overall appearance of the “indulgent” breakfast higher than the “sensible” breakfast (mean difference: 5.00 [95% CI: 0.71, 9.30]; *p* = 0.024), but felt less healthy when consuming the “indulgent” breakfast (mean difference: −13.17 [95% CI: −18.75, −7.60]; *p* <0.001). Participants did not rate the breakfasts differently according to their taste, smell, overall palatability or enjoyment.

### 3.3. Self-Reported Appetite 

Table 4 shows the changes in self-reported appetite measures from baseline to anticipatory (BL-ANT), anticipatory to post-consumption (ANT-POST) and baseline to post-consumption (BL-POST) according to breakfast condition (indulgent vs. sensible). Participants reported a significantly higher mean change in fullness score (i.e., feel fuller) for the “indulgent” breakfast than the “sensible” breakfast from ANT to POST (mean change difference: 7.19 [95% CI: −0.73, 13.6]; *p* = 0.030). This relationship was not evident from BL to POST, but did approach significance from BL to ANT, although in the opposite direction (mean change difference: −2.89 [95% CI: −5.90, 0.12]; *p* = 0.059). The mean reduction in the strength of desire to eat from BL to POST between the breakfasts also approached significance (mean change difference: −6.77 [95% CI: −13.8, 0.24]; *p* = 0.058). There were no significant mean changes observed for the other appetite measures between the breakfasts at any of the time points.

When the sample was stratified by level of restrained eating behaviour, those with a low restrained eating score reported a significantly lower mean change in fullness score (i.e., feel less full) from baseline to anticipatory for the “indulgent” breakfast than the “sensible” breakfast (mean change difference: −5.52 [95% CI: −9.65, −1.39]; *p* = 0.011). During the same time period, those classified as having a high restrained eating score reported a significantly lower mean change in their strength of desire to eat for the ‘indulgent’ breakfast compared with the “sensible” breakfast (mean change difference: −5.87 [95% CI: −11.05, −0.68]; *p* = 0.028). There were no significant mean changes observed for the other appetite measures between the breakfasts in the low or high restrained eating groups at any of the time points (Supplementary Table S1).

### 3.4. Gastrointestinal Hormones

Table 5 shows the changes in acylated ghrelin, GLP-1 and PYY measurements from baseline to anticipatory (BL-ANT), anticipatory to post-consumption (ANT-POST) and baseline to post-consumption (BL-POST) according to breakfast condition (indulgent vs. sensible). There were no significant differences in gut hormone levels between the breakfasts at any of the time points, and this was also observed when the sample was stratified by level of restrained eating behaviour (results not shown).

## 4. Discussion

Evidence is mounting that a person’s ideas and expectations about the food they are eating can alter taste, preference and consumption. Previous research has also suggested that this may alter the physiological response to food by accelerating the fall in post-prandial ghrelin [18]. If these effects on perceived satiety and gut hormone physiology could be demonstrated across a broad range of foods, food labelling could potentially be manipulated to maximise perceived and physiological satiation.

Whilst it is appreciated that this study was exploratory in nature and involved testing at multiple timepoints, some useful insights were gained. This study demonstrated a significant increase in self-reported fullness after consumption of the “indulgent” breakfast versus the “sensible” breakfast which is what might be expected if the actual calorie and macronutrient content of the two products differed. The increase in self-reported fullness in the current study is likely secondary to the participants’ perceptions of the products based on the food labels. It is clear that participants had formed perceptions of the breakfast products healthiness both prior to, and during, product consumption; prior to consumption participants rated the indulgent breakfast as being less healthy and they reported feeling less healthy whilst consuming it. However, it is also possible that these effects are secondary to response bias, with participants feeling that they ought to feel fuller after the higher calorie product and reporting fullness as such. It is recognised that the participant population had a high level of education. Education has been shown to influence perception of taste and satiety from food labelling [8] and has been associated with increased interest in and use of food labels [28,29]. It would be useful therefore to explore whether the effects observed in this study are replicable across different levels of education.

Some limitations of the study design are acknowledged. Firstly, the time and nature of the last meal the preceding evening was not specified which may have affected baseline measures of hunger. Furthermore, it is not known whether the participants were regular consumers of breakfast, or if they were familiar with the particular kind of breakfast product. Familiarity with the breakfast product may be associated with pre-existing ideas of taste and health and this may have affected subjective measures of hunger. The authors also acknowledge that there may be carry-over effects in cross-over studies of this kind that mean the effect of the manipulation may be underestimated [30]. However, the order of the breakfast products was randomly allocated to ensure the presentation of test conditions was counterbalanced to neutralise possible learning effects.

In a study by Crum et al. (2011) there was a rise in ghrelin in anticipation of an “indulgent” milkshake when compared to anticipation of an identical milkshake described as “sensible”. This was followed by a significantly greater rate of reduction in ghrelin following consumption [18]. In the current study, there was no difference in circulating levels of acylated ghrelin between the breakfasts at any time point. There is some evidence that ghrelin rises physiologically in anticipation of an expected meal, rather than solely being responsible for meal initiation [31]. The mechanisms driving this are not fully understood and it is possible that the expected size or macronutrient composition of the anticipated meal may modify this effect. It is therefore possible that different food products could elicit different responses, with health claims further altering the response. There were no significant differences in subjective hunger ratings between the two groups in this study or in the study by Crum et al. This supports the notion that the ghrelin rise seen in the study by Crum et al. could be due to meal anticipation rather than changes in the metabolic parameters which drive hunger.

Although this study demonstrated a significant increase in self-reported fullness after consumption of the “indulgent” breakfast versus the “sensible” breakfast, there were no significant differences in the circulating levels of GLP-1 or PYY between the breakfasts at any time point. This suggests that the increased perceived satiety following ingestion of the supposed “indulgent’ breakfast is independent of anorectic gut hormone signalling, possibly occurring via manipulation of the hedonistic rather than homeostatic pathways involved in appetite regulation. Individuals who report placing high importance on taste have both increased consumption and self-reported satiety on consumption of a salad labelled as being “hearty” versus a salad labelled as being “healthy” [32]. This contrasts with individuals who place low importance on taste who eat less but report similar satiety scores when eating the “hearty” versus the “healthy” salad. This increased susceptibility to food labelling demonstrated by individuals who place high importance on taste supports the role of non-homeostatic regulatory pathways in mediating the effect of food labelling on satiety.

Prior to consumption of the breakfast products, participants rated the indulgent option as being more appealing. During consumption, however, participants did not report any difference in the taste, smell, overall palatability or enjoyment between the two breakfast items. This finding is contrary to studies which show that food labelled and described as tasty has higher taste scores following consumption compared to the same food described as healthy [3]. It is possible that suggestion from labelling associated with a differential in taste has more of an effect on the non-homeostatic regulation of food preference than suggested differentials in health.

Evidence suggests that labelling a food as healthy encourages restrained eaters to eat more [21]. In this study restrained eaters did not report any differences between the breakfasts with regard to appetite change from anticipatory to post-consumption. They did, however, report a significantly lower mean change in their strength of desire to eat in anticipation of the “indulgent” breakfast compared to the “sensible” breakfast. The sensible breakfast was perhaps a more appealing option for restrained eaters due to its apparent lower calorie, fat and sugar content.

Whilst in the current study food labelling influenced the sensation of fullness, the physiological state of eating inhibition, it did not impact other appetite sensations including hunger. It is possible that the timing between consumption and completing the self-reported appetite measures was insufficient for the processing of satiety signals. It is also possible that the study was inadequately powered to detect the changes in appetite measures. As this was an exploratory study based on a similar experiment [18], a formal power calculation was not used. The effect sizes on self-reported fullness demonstrated in this study and in other studies cited here are small. It is possible that other studies have been conducted which have demonstrated no such effects and remain unpublished. The clinical relevance of the results are therefore uncertain.

The increase in self-reported fullness following consumption of the “indulgent” breakfast compared with the “sensible” breakfast is likely secondary to the participants’ perceptions of the products based on the food labels. However, the effect sizes were small and future studies could include a positive control condition where the actual energy content of the product is manipulated in an oro-sensory matched manner to determine relative effect sizes. Evidence suggests that nutrient and health claims used for marketing purposes such as ‘lower in fat’ could actually encourage people to eat more [14]. This could be mediated by calorie underestimation and subsequent overconsumption. The current study manipulated participants’ perception of both calorie content and hedonic expectations. It would be beneficial to evaluate the effect of manipulation of perception of energy content alone on reported satiety measures as this may be more easily incorporated into current product calorie labelling strategies. Obesity rates are rising despite simultaneous increases in availability and sales of ‘healthy’ food products. Further study is needed to determine the effects of nutritional labelling on levels of food consumption and risk of weight gain.

As discussed above, participants rated the indulgent option as being more appealing prior to consumption but reported no difference in overall palatability during consumption. Other studies have found that taste-focussed labelling has more of an effect on food choice than health-focussed labelling [3]. Further research is needed to identify particular labelling ‘triggers’ that promote preference for that particular food. This could be particularly relevant for public health initiatives in promoting healthy food choices. If suggested taste is indeed a superior influencer of food choice, research into the cognitive pathways governing this could be of further interest. Given the evidence suggesting that restrained eaters associate unhealthy food items with superior taste, this could be of particular importance for this sub-group.

The current study found no difference in the magnitude of change of gut hormone levels between the two breakfasts across the time points of the study, suggesting that the alterations in fullness scores observed are likely not mediated by gut hormone physiology. These findings are in contrast to a previous similar study that reported a relative preprandial rise in ghrelin followed by a steeper post-prandial decline [18]. It is possible that this ghrelin rise could be due to meal anticipation and further study of the variance in pre-prandial ghrelin release with different expectations of meal size and macronutrient content would be of interest. In the current study there were no significant differences in ghrelin levels at any time point when stratified by levels of restrained eating behaviour. However, previous studies have shown that restrained eaters have significantly higher ghrelin levels than non-restrained eaters, both in the fasting state and when consuming a palatable milkshake [20,33]. It is possible that the sample sizes in this study were insufficient to detect significant differences within the restrained eaters and further study is warranted to examine the differences in gut hormone secretion in those with restrained eating patterns.

## 5. Conclusions

This experimental study demonstrated that satiation, as measured by self-reported fullness, varies according to the beliefs and expectations about the food being consumed, and that the changes to satiety seen with health and nutrient-focussed food labelling are likely not mediated by alterations in gut hormone release. Given the conflicting evidence regarding the effects of food labelling on appetite, further study is required to determine whether food labelling can meaningfully impact satiety. Furthermore, the longer-term effects on food intake and subsequent body weight should be elucidated. 

## Figures and Tables

**Table 1:** Nutritional information for the breakfast product.

	Kcal	Fat (g)	Carbohydrate (g)	Protein (g)
**Natural yoghurt (150g)**	123	6.8	8.4	7.7
**Granola (50g)**	209	5.6	32.8	5.1
**Compote (30g)**	48	0.3	10.5	0.2
**Total (230g)**	380	12.7	51.7	13

**Table 2:** Sample characteristics.

Sample Characteristics		
Total sample *n* (%)		50 (100)
Sex *n* (%)	Female	33 (66)
Age (years) mean (SD)		30.1 (10.4)
Geographical location *n* (%)	Urban	31 (62)
	Suburban	13 (26)
	Rural	6 (12)
^a^ Occupational classification *n* (%)	Higher managerial, admin & professional	11 (22)
	Intermediate occupations	8 (16)
	Routine & manual occupations	0 (0)
	Student (undergraduate/postgraduate)	31 (62)
Education level (years) mean (SD)		18.4 (3.1)
BMI (kg/m^2^) mean (SD)		24.6 (3.6)
BMI categories *n* (%)	Normal weight (18.5–24.9 kg/m^2^)	31 (62)
	Overweight (25–29.9 kg/m^2^)	14 (28)
	Obese (>30 kg/m^2^)	5 (10)
How often read nutrition information on food labels *n* (%)	Often/always	29 (60.4)
	Occasionally/rarely	18 (37.5)
	Never	1 (2.1)
How often read health claims on food labels *n* (%)	Often/always	21 (43.8)
	Occasionally/rarely	18 (45.8)
	Never	5 (10.4)

Data presented as mean (SD) for continuous variables and frequency (%) for categorical variables. ^a^ Occupational classification analysed using NS-SEC.

**Table 3:** The effect of breakfast condition (indulgent vs. sensible) on breakfast package rating and palatability rating.

	Indulgent Breakfast		Sensible Breakfast			
Visual Analogue Scales	Sample Size	Mean (SD)	Sample Size	Mean (SD)	Mean Difference ^a^ (95% CI)	*p*
Breakfast package rating						
Appeal	48	87.12 (10.5)	48	69.7 (19.3)	17.3 (11.6, 23.0)	<0.001
Perceived healthiness	48	45.8 (21.1)	48	70.5 (13.6)	−24.8 (−31.6, −17.9)	<0.001
Palatability rating						
Breakfast (food) appearance	48	84.3 (12.4)	48	79.1 (13.8)	5.0 (0.7, 9.3)	0.024
Smell of the breakfast	48	77.1 (16.2)	48	75.8 (13.7)	1.5 (−2.4, 5.3)	0.455
Taste of the Breakfast	48	80.0 (13.0)	48	78.3 (13.2)	1.65 (−2.8, 6.1)	0.462
Overall palatability	48	79.4 (16.1)	48	80.2 (13.8)	−0.7 (−6.0, 4.5)	0.777
Enjoyed the breakfast	47	80.2 (16.1)	47	77.3 (15.6)	2.8 (−3.0, 8.6)	0.336
Healthy feeling while eating breakfast	47	58.9 (19.8)	47	72.2 (13.3)	−13.2 (−18.8, −7.6)	<0.001

Data are presented as mean (SD), and ^a^ mean difference (95% CI) in VAS scores between breakfast conditions (indulgent vs. sensible) adjusted for the period effect. Data analysed using paired sample *t*-tests adjusting for period effect.

**Table 4:** Changes in self-reported appetite measures according to breakfast condition (indulgent vs. sensible).

			Indulgent Breakfast	Sensible Breakfast		
	Time Point	Sample Size	Mean Change (SD)	Mean Change (SD)	Mean Difference (95% CI)	*p*
Hunger	BL	48	63.80 (19.31) *	62.72 (76.05) *	-	-
	BL-ANT	48	15.29 (14.03	13. 33 (17.86)	1.73 (−4.40, 7.86)	0.572
	ANT-POST	48	−55.28 (22.48)	−50.09 (22.61)	−5.06 (−11.4, 1.27)	0.114
	BL-POST	48	−39.99 (23.55)	−36.76 (22.43)	−3.33 (−9.48, 2.83)	0.282
Satisfied	BL	48	33.56 (22.17) *	34.29 (21.43) *	-	-
	BL-ANT	48	−7.33 (15.08)	−8.14 (15.75)	0.99 (−4.87, 6.85)	0.736
	ANT-POST	48	51.66 (25.09)	48.32 (24.78)	3.40 (−2.59, 9.39)	0.259
	BL-POST	48	44.32 (27.45)	40.19 (25.14)	4.39 (−2.99, 11.8)	0.237
Fullness	BL	48	21.96 (16.32) *	22.24 (19.67) *	-	-
	BL-ANT	48	−5.73 (9.70)	−2.94 (14.56)	−2.89 (−5.90, 0.12)	0.059
	ANT-POST	48	57.18 (21.66)	49.96 (22.90)	7.19 (0.73, 13.6)	0.030
	BL-POST	48	51.45 (21.19)	47.03 (21.45)	4.30 (−2.04, 10.6)	0.179
^a^ Quantity	BL	48	62.98 (16.49) *	62.49 (15.62)	-	-
	BL-ANT	48	10.70 (12.10)	9.95 (11.23)	0.63 (−4.35, 5.60)	0.801
	ANT-POST	48	−46.21 (19.03)	−45.18 (19.59)	−1.05 (−5.70 3.60)	0.653
	BL-POST	48	−35.51 (17.31)	−35.23 (19.31)	−0.42 (−5.96, 5.11)	0.879
^b^ Desire	BL	48	67.98 (21.66) *	65.21 (21.16) *	-	-
strength	BL-ANT	48	10.53 (14.21)	12.32 (14.58)	−1.94 (−6.01, 2.12)	0.340
	ANT-POST	48	−52.45 (23.42)	−47.53 (21.19)	−4.82 (−11.9, 2.24)	0.176
	BL-POST	48	−41.92 (26.82)	−35.21 (24.79)	−6.77 (−13.8, 0.24)	0.058

Data are presented as baseline mean (SD)*, mean change (SD), and mean change difference (95% CI) in appetite measures between breakfast conditions (indulgent vs. sensible), adjusted for the period effect, at baseline (BL), from baseline to anticipatory (BL-ANT), from anticipatory to post consumption (ANT-POST) and from baseline to post-consumption (BL-POST). Data analysed using paired sample *t*-tests adjusting for period effect. ^a^ Quantity—how much do you think you could (or would want to) eat right now? ^b^ Desire strength—how strong is your desire to eat?

**Table 5:** The effect of breakfast condition (indulgent vs. sensible) on changes in gut hormones.

			Indulgent Breakfast	Sensible Breakfast		
Gut Hormone	Time Point	Sample Size	Mean Change (SD)	Mean Change (SD)	Mean Difference (95% CI)	*p*
Acylated	BL		32.46 (41.86) *	32.59 (44.83) *	-	-
ghrelin	BL-ANT	38	3.04 (13.10)	4.66 (15.65)	−0.86 (−5.02, 3.31)	0.680
pmol/L	ANT-POST	38	−8.38 (10.88)	−10.36 (15.8)	1.68 (−3.96, 7.32)	0.549
	BL-POST	38	−5.35 (14.58)	−5.70 (11.21)	0.83 (−5.17, 6.83)	0.781
PYY pmol/L	BL		36.66 (31.45) *	34.00 (33.21) *	-	-
	BL-ANT	40	−2.15 (8.99)	−2.13 (8.96)	0.01 (−4.29, 4.32)	0.995
	ANT-POST	40	6.23 (10.78)	6.74 (10.49)	−0.65 (−5.09, 3.79)	0.768
	BL-POST	40	4.01 (11.93)	4.74 (8.35)	−0.64 (−5.79, 4.51)	0.803
GLP-1 pmol/L	BL		40.35 (10.83) *	40.46 (11.89) *	-	-
	BL-ANT	44	0.10 (5.96)	0.17 (5.59)	−0.14 (−2.68, 2.41)	0.913
	ANT-POST	44	14.34 (11.67)	14.14 (11.25)	0.41 (−3.93, 4.76)	0.848
	BL-POST	44	14.44 (11.46)	14.31 (11.29)	0.28 (−4.33, 4.88)	0.904

Data are presented as baseline mean (SD)*, mean change (SD), and mean change difference (95% CI) in gut hormone levels between breakfast conditions (indulgent vs. sensible) at baseline (BL), from baseline to anticipatory (BL-ANT), from anticipatory to post consumption (ANT-POST) and from baseline to post-consumption (BL-POST). Data analysed using paired sample *t*-tests adjusting for period effect.

## Data Availability

Data described in the manuscript can be shared publicly upon reasonable justified request.

## Supplementary Materials

The following supporting information can be downloaded at https://www.mdpi.com/article/10.3390/nu14235100/s1, Table S1: Changes in self-reported appetite measures according to breakfast condition (indulgent vs. sensible) and level of restrained eating (high vs. low).
