# Supplementary material for: The Power of Suggestion: Subjective Satiety Is Affected by Nutrient and Health-Focused Food Labelling with No Effect on Physiological Gut Hormone Release
Source: Nutrients. 2022 Dec 1;14(23):5100. doi: 10.3390/nu14235100 (PMC9740488; doi:10.3390/nu14235100)
Supplement: Supplementary file 1 [file nutrients-14-05100-s001.zip › nutrients-2059280-supplementary.pdf]

Supplementary table S1. Changes in self–reported appetite measures according to breakfast condition (indulgent *vs*. sensible) and level of restrained eating (high *vs*. low)

	Time point	Sample size	Indulgent Breakfast Mean change (SD)	Sensible Breakfast Mean change (SD)	Mean difference (95% CI)	P
**High restrained eating score**						
**Hunger**	BL	26	68.71 (18.35)*	63.17 (18.38)*	–	–
	BL–ANT	26	10.92 (12.55)	12.56 (17.61)	−1.10 (−9.89, 7.70)	0.799
	ANT–POST	26	−56.81 (21.46)	−51.96 (22.87)	−5.50 (−15.28, 4.29)	0.258
	BL–POST	26	−45.88 (22.35)	−39.40 (19.90)	−6.59 (−16.27, 3.08)	0.172
**Satisfied**	BL	26	30.94 (21.64)*	36.69 (23.81)*	–	
	BL–ANT	26	−5.71 (15.72)	−10.40 (14.54)	4.27 (−4.13, 12.67)	0.305
	ANT–POST	26	53.40 (26.41)	50.88 (24.28)	2.60 (−6.10, 11.30)	0.543
	BL–POST	26	47.69 (27.45)	40.48 (28.91)	6.87 (−3.64, 17.38)	0.190
**Fullness**	BL	26	18.96 (14.29)*	23.69 (19.39)*	–	–
	BL–ANT	26	−4.08 (8.77)	−3.35 (13.62)	−0.45 (−4.90, 4.00)	0.837
	ANT–POST	26	59.94 (18.75)	51.42 (23.27)	8.48 (−2.35, 19.31)	0.119
	BL–POST	26	55.87 (18.21)	48.07 (22.15)	8.03 (−1.98, 18.05)	0.111
**Quantity**	BL	26	62.31 (17.36)*	61.94 (16.33)*	–	–
	BL–ANT	26	9.98 (12.61)	9.04 (11.48)	1.08 (−6.53, 8.69)	0.773
	ANT–POST	26	−46.96 (19.31)	−46.12 (18.68)	−0.87 (−7.41, 5.67)	0.786
	BL–POST	26	−36.98 (18.17)	−37.08 (19.72)	0.21 (−7.62, 8.04)	0.957
**Desire strength**	BL	26	69.52 (22.10)*	63.58 (22.50)*	–	–
	BL–ANT	26	7.96 (11.78)	13.98 (14.05)	−5.87 (−11.05, −0.68)	0.028
	ANT–POST	26	−53.85 (22.21)	−52.48 (19.96)	−1.66 (−12.34, 9.02)	0.752
	BL–POST	26	−45.88 (23.73)	−38.50 (25.38)	−7.53 (−17.58, 2.52)	0.135
**Low restrained eating score**						

**Hunger**	BL	22	58.00 (19.20)*	62.18 (21.38)*	–	–
	BL−ANT	22	20.45 (14.20)	14.25 (18.53)	5.74 (−3.54, 15.02)	0.212
	ANT−POST	22	−53.48 (24.00)	−47.89 (22.62)	−6.23 (−14.17, 1.72)	0.118
	BL−POST	22	−33.02 (23.52)	−33.64 (25.22)	−0.49 (−8.19, 7.22)	0.896
**Satisfied**	BL	22	36.66 (22.89)*	31.46 (18.36)*	–	–
	BL−ANT	22	−9.25 (14.41)	−5.45 (17.02)	−3.42 (−12.24, 5.41)	0.429
	ANT−POST	22	49.59 (23.89)	45.30 (25.58)	5.24 (−3.79, 14.28)	0.240
	BL−POST	22	40.34 (27.54)	39.84 (20.46)	1.83 (−9.75, 13.40)	0.746
**Fullness**	BL	22	25.50 (18.13)*	20.52 (20.31)*	–	–
	BL−ANT	22	−7.68 (10.58)	−2.45 (15.92)	−5.52 (−9.65, 1.39)	0.011
	ANT−POST	22	53.91 (24.71)	48.25 (22.87)	5.24 (−2.20, 12.68)	0.157
	BL−POST	22	46.23 (23.60)	45.80 (21.04)	−0.28 (−8.38, 7.83)	0.944
[a]**Quantity**	BL	22	63.77 (15.77)*	63.14 (15.10)*	–	–
	BL−ANT	22	11.55 (11.70)	11.02 (11.09)	−0.33 (−7.42, 6.77)	0.924
	ANT−POST	22	−45.32 (19.10)	−44.07 (21.00)	−1.49 (−8.98, 6.01)	0.684
	BL−POST	22	−33.77 (16.49)	−33.05 (19.04)	−1.81 (−10.55, 6.93)	0.670
[b]**Desire strength**	BL	22	66.16 (21.50)*	67.14 (19.80)*	–	–
	BL−ANT	22	13.57 (16.40)	10.36 (15.28)	2.38 (−4.13, 8.89)	0.455
	ANT−POST	22	−50.80 (25.19)	−41.68 (21.56)	−9.25 (−19.18, 0.68)	0.066
	BL−POST	22	−37.23 (29.94)	−31.32 (24.06)	−6.87 (−17.66, 3.92)	0.199

Data are presented as *baseline mean (SD), mean change (SD), and mean change difference (95% CI) in appetite measures between breakfast conditions (indulgent *vs.* sensible) adjusted for the period effect. Data analysed using paired sample t−tests adjusting for period effect, at baseline (BL), from baseline to anticipatory (BL−ANT), from anticipatory to post consumption (ANT−POST) and from baseline to post−consumption (BL−POST). [a]Quantity – how much do you think you could (or would want to) eat right now? [b]Desire strength – how strong is your desire to eat?
